# Characterizing neonatal community-acquired invasive fungal infections: Clinical profiles, pathogens, and underlying conditions

**DOI:** 10.1097/MD.0000000000042921

**Published:** 2025-06-20

**Authors:** Chunfang Gao, Li Wang, Lingkong Zeng, Xuwei Tao

**Affiliations:** a Department of Neonatology, Wuhan Children’s Hospital of Tongji Medical College, Huazhong University of Science & Technology, Wuhan, China.

**Keywords:** acquired, clinical features, community, invasive fungal infection, neonate

## Abstract

**Objective:**

To characterize the clinical presentation and diagnostic indicators of neonatal community-acquired invasive fungal infections and establish a systematic approach for early identification and management.

**Methods:**

This study retrospectively reviewed the medical records of neonates discharged from the neonatal department between 1/1/2019 and 1/12/2023. Cases were identified based on the primary or first diagnosis using the International Classification of Diseases, 9th Revision codes. A total of 6 patients were included and comprehensive clinical data were analyzed. A systematic literature review (PubMed/Embase, 2000–2023) was conducted to contextualize findings.

**Results:**

6 neonates, including 5 boys, weighed 2700 to 4480 g, hospitalized at the age of 15 to 26 days. Key findings included: Patients had nonspecific respiratory symptoms, such as cough, tachypnea, and fever; the auxiliary examination showed positive β-ᴅ-glucan (cases 1, 4, 5) and galactomannan test (cases 2, 3, 4), and all cases had elevated CD4+/CD8 + ratio; multifocal consolidations (cases 2, 3) with halo sign evolution on serial imaging; pathogen spectrum were: *Aspergillus flavus* (3/6), *Candida spp.* (2/6), *Lichtheimia corymbifera* (1/6); antifungal treatments achieved clinical resolution in all cases (median duration 24 days), with sustained remission at 3-month follow-up; underlying immunometabolic disorders identified post-diagnosis in 83% (5/6) cases, including chronic granulomatous disease, lupus syndrome, and methylmalonic acidemia.

**Conclusion:**

Neonatal community-acquired invasive fungal infections represents a critical diagnostic challenge requiring for its low incidence and nonspecific clinical features. Diagnosis mainly based on the fungi-culture. Appropriate use of antifungi medication can lead to a better outcome. Moreover, suspicion of the latent diseases which can cause immune and metabolic dysfunction would be benefit for improving prognosis.

## 1. Introduction

The global burden of invasive fungal infections (IFIs) has has significantly increased in recent decades, with incidence rates in immunocompromised populations rising 1.5- to 3-fold between 2010 and 2022.^[1]^ This trend is closely linked to modern medical advancements, such as the prolonged use of immunosuppressive therapies, increasing rates of hematopoietic stem cell transplantation, and improving survival of extremely premature neonates.^[2]^ The neonatal population is particularly affected, with data from the Global Action Fund for Fungal Infections showing a 27% increase in invasive candidiasis among very low birth weight infants since 2015.^[3]^ IFIs, which are deep-seated or bloodstream infections caused by *Candida spp.* or other emerging molds, are associated with severe clinical outcomes. Mortality rates in cases of disseminated infection can reach 20% to 40%.^[4]^ While hospital-acquired fungal infections in neonatal intensive care units have been extensively studied, especially in preterm infants with a gestational age of <28 weeks and a birth weight of <1000 g, there remains a critical knowledge gap concerning community-acquired invasive fungal infections (CA-IFIs) in term neonates.^[5]^ Diagnostic frameworks, primarily developed for Neonatal Intensive Care Unit (NICU) populations, may not adequately address the unique pathophysiology of CA-IFIs.

At our tertiary perinatal center, we identified 6 cases of culture-proven CA-IFIs between 2019 and 2023. These cases defied conventional risk stratification models and exhibited delayed diagnoses due to atypical presentations. This case series aims to delineate the evolving clinical phenotypes of neonatal CA-IFIs and to provide insights for the development of targeted surveillance protocols for this vulnerable population.

## 2. Patients and methods

We extracted International Classification of Diseases, 9th Revision codes related to fungal infections from hospital discharge records in NICU between 1/1/2019 and 1/12/2023. A total of 6 patients were included. A proven or probable IFI was defined according to the revised European Organization for Research and Treatment of Cancer and the Mycoses Study Group Education and Research Consortium (EORTC/MSGERC) criteria.^[6]^ Six patients who met the diagnostic criteria for IFIs and had confirmed community-acquired infections were included in the study.

For each patient, the following data were collected:

1. Demographic and background information: Gender, gestational age, age at symptom onset, prenatal and postnatal history, previous medical history, family history, and contact history.
2. Clinical data: Clinical manifestations, complications, therapeutic protocols (including medications and duration), and outcomes; laboratory tests included complete blood count, biochemical parameters, microbiological cultures, and immunological assessments; radiological examinations included chest X-ray, chest CT, and head MRI.
3. Follow-up information: Follow-up was conducted via weekly telephone consultations or outpatient visits for 3 months post-discharge.

## 3. Ethical considerations

This study was retrospective; all the data were collected as part of the routine procedure, and the analysis did not influence patient management. This study was conducted in accordance with the principles of the Declaration of Helsinki. This study was approved by the Ethics Committee of Wuhan Women and Children’s Medical Care Center (2021R050-E01), and the parents signed an informed consent form.

## 4. Results

A total of 6 neonates, 5 of whom were male, were delivered at term with gestational ages ranging from 37^+3^ weeks to 40^+5^ weeks, and birth weights between 2560 and 3660 g. None of the infants had prenatal abnormalities or documented familial hereditary diseases. All neonates were raised at home and exclusively breastfed, with no prior history of illness. Case 4 had a known history of poultry contact.

Symptom onset in these patients occurred between 15 and 26 days of age, primarily characterized by infection-related complaints such as fever and respiratory symptoms. The duration of fever before admission ranged from 1 to 4 days among cases 1, 3, 4, and 6, with peak temperatures between 38°C and 39.4°C. Prior to admission to our center, cases 1, 3, and 6 had received ineffective antibiotic treatment either in other hospitals or outpatient settings. Throughout their illness, patients exhibited varying degrees of fever, cough, nasal congestion, and shortness of breath. Additionally, cases 2, 5, and 6 experienced vomiting episodes, while case 4 presented with irritability and loss of appetite (Table 1). Upon admission, physical examination revealed infection-related findings, although none of the patients exhibited obvious signs of septic toxicity. Atypical symptoms included diminished mental status, minor petechial rashes on the skin, and coarse breath sounds with audible rales in both lungs. Notably, the cardiovascular, digestive, urinary, reproductive, and nervous systems showed no significant abnormalities.

**Table 1:** Clinical characteristics of 6 cases.

	Gender	Apgar score	Gestational age	Weight(g, birth/onset)	Onset (d)	Complaint	Symptoms during the course of the disease	Treatments	Days of stay	Outcome
Case 1	Male	9-10-10	37 + 5	3015/4000	24	Fever for 3 d	Fever, cough	*Ceftazidime* for 6 d (other hospital 3 d), *Fluconazole* (intravenous for 16 d, oral for 2 wk)	18	cured
Case 2	Female	9-10-10	37 + 3	2560/2700	21	Shortness of breath for 4 d	Shortness of breath, cough, vomit	*Cefuroxime* for 3 d, *Voriconazole*(intravenous for 21 d, oral for 1wk)	25	Bettered, slightly cough for nearly 2 wk after charge
Case 3	Male	9-9-10	38 + 3	3550/3890	20	Fever for 4 d	*Fever, cough, nasal obstruction*	*Cefuroxime* for 6 d (3 d in outpatient), *Voriconazole*(intravenous for 21 d, oral for 1 wk)	25	Bettered, slightly cough for nearly 1 wk after charge
Case 4	Male	9-10-10	37 + 3	2750/3280	20	Fever for 1 d	*Fever, cough, irritation, loss of appetite*	*Ceftazidime* for 4 d, *Voriconazole*(intravenous for 2 wk, oral for 2 wk)	20	Cured
Case 5	Male	9-10-10	40 + 5	3660/4480	26	Cough for 1 wk	*Cough, nasal obstruction, vomit*	*Ceftazidime* for 4 d (3 d outpatient), *Azithromycin* for 2 wk, *Voriconazole*(intravenous for 2 wk, oral for 2 wk)	22	Bettered, slightly cough for 2 wk after charge
Case 6	Male	9-10-10	38 + 2	3270/3540	15	Fever for 3 d	*Fever, cough*	*Cefuroxime f*or 5 d (2 d in outpatient), *Fluconazole* (intravenous for 14 d, oral for 1 wk)	18	Cured

During hospitalization, a comprehensive set of laboratory assays was performed. The complete blood count revealed abnormalities in platelet count and monocyte ratio. Platelet counts ranged from 138–637 × 10⁹/L across cases 1 to 5, while the monocyte ratio was elevated in all cases, ranging from 13% to 19.2%. Apart from an increased leukocyte count in case 3, erythrocyte counts, hemoglobin levels, and hematocrit values were within normal limits in all cases. Hypersensitive C-reactive protein (hs-CRP) levels were significantly elevated in all cases, ranging from 18.9 to 181 mg/L. Immune function tests showed notable changes: the IgE levels were approximately 8 times higher than normal, and the CD4/CD8 ratio was elevated in all cases, ranging from 2.17 to 4.97. Lumbar puncture and cerebrospinal fluid (CSF) examination were performed in all patients, with only case 4 showing an increased leukocyte count in the CSF; biochemical parameters were unremarkable. β-ᴅ-glucan levels were elevated in all cases, ranging from 221 to 316 pg/mL, and the galactomannan (GM) test was positive in cases 2, 3, and 4.

**Figure 1.**
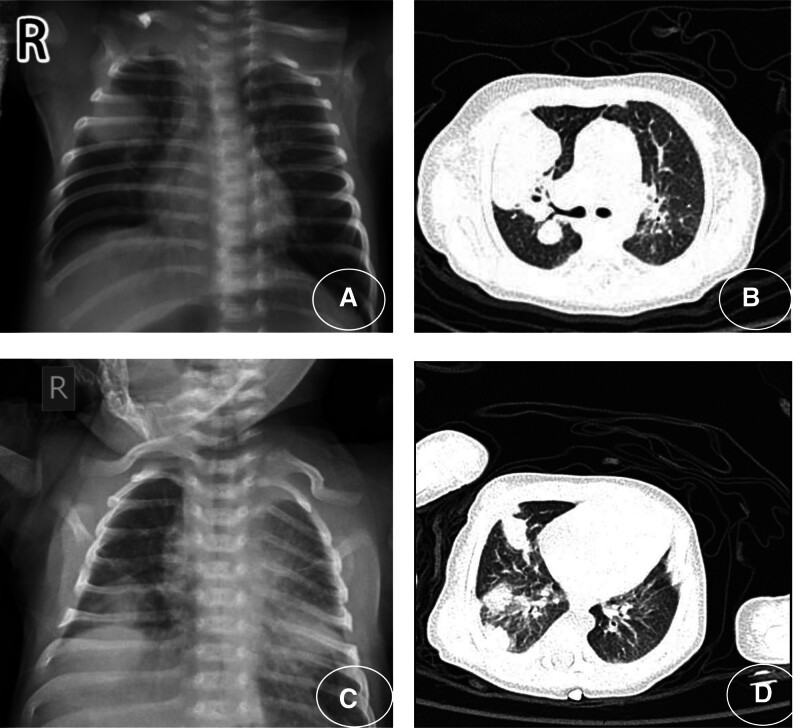
Pulmonary radiology (lung X-ray and computed tomography): case 2 (A and B) and case 3 (C and D).

**Figure 2.**
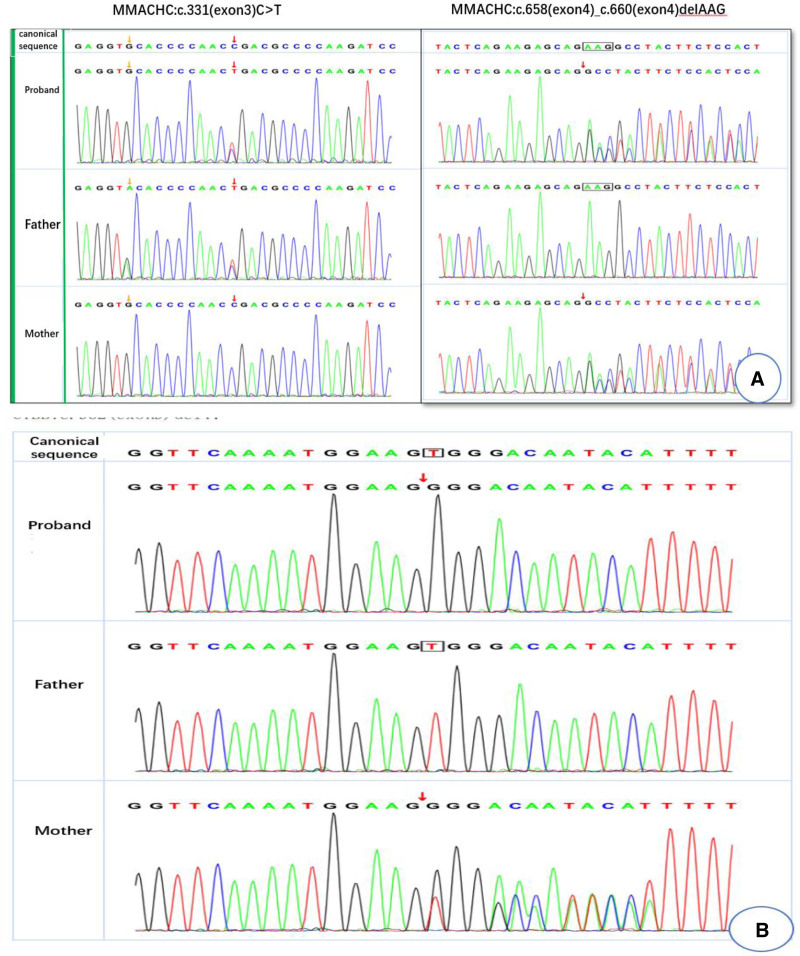
Results of the whole-exome genome test. (A) Case 2: c.331 C > T; c.658-c.660delAAG. (B) Case 3: CYBB:c.962(exon9)delT.

Microbiological cultures from peripheral blood, bronchoalveolar lavage fluid, and cerebrospinal fluid identified the pathogens in each case: *Candida guilliermondii* (case 1, peripheral blood); *A flavus* (cases 2 and 3, bronchoalveolar lavage fluid); *L corymbifera* and Enterovirus (case 4, peripheral blood and cerebrospinal fluid); *A flavus* and *Chlamydia pneumoniae* (case 5, peripheral blood and bronchoalveolar lavage fluid); and *Candida tropicalis* (case 6, peripheral blood). These findings are summarized in Table 2. X-rays and CT scans revealed dense opacities in cases 2 and 3 (Fig. 1), while other cases showed signs of interstitial lung disease. After confirming IFI, fundus examination, brain MRI, and organic color Doppler ultrasonography were performed, with no abnormalities detected. Further testing revealed that case 1 was positive for antinuclear antibodies and was subsequently diagnosed with neonatal lupus syndrome. Whole-exome sequencing of case 2 identified methylmalonic acidemia with the genotype c.331 C > T; c.658-c.660delAAG. Case 3 was diagnosed with chronic granulomatous disease, harboring a variant of the CYBB gene (c.962 del T; Fig. 2).

**Table 2:** Auxiliary examinations of 6 neonates at the onset point.

	Reference	Case 1	Case 2	Case 3	Case 4	Case 5	Case 6
Blood routine test							
WBC	5–20 (×10^9^/L)	17.01	15.1	23.3	5.18	13.66	6.81
RBC	3.5–5.4 (×10^12^/L)	3.9	4.1	3.97	3.15	3.82	3.54
PLT	242–378 (×10^9^/L)	484	138	637	418	199	249
HGB	115–135 (g/L)	113	106	115	119	121	116
HCT	31–52 (%)	56.6	47.7	58.7	51.1	39.4	34.5
M (%)	1–12 (%)	13	19.2	16.2	16.4	19	14.7
Hs-CRP	0–3 (mg/L)	181	21.7	117	18.9	20.8	65.7
Immune function							
IgG	4–14.8 (g/L)	6.46	5.52	6.19	4.57	9.36	5.65
IgA	0–0.22 (g/L)	0.24	0.59	0.27	0.42	0.39	0.21
IgM	0.05–0.3 (g/L)	0.5	0.46	0.52	0.26	0.27	0.23
C3	0.6–1 (g/L)	1.59	0.96	1.16	0.73	1.02	0.79
IgE	0–15 (g/L)	10.2	12.9	109	6	5	2.21
C4	0.1–0.36 (g/L)	0.29	0.16	0.25	0.28	0.18	0.25
Cerebrospinal fluid examinations							
WBC	0–15 (×10^6^/L)	4	4	5	216	4	6
PRO	0.12–0.6 (g/L)	0.7	0.9	0.53	0.53	0.35	0.47
CL	118–128 (mmol/L)	113.6	121.3	123.4	119.3	119.7	120.4
GLU	2.2–3.9 (mmol/L)	2.58	2.06	2.96	2.61	2.76	3.04
TBNK							
CD3-t	805–4459 (/μL)	3191	2735	4484	1777	2731	1906
CD8-t	314–2080 (/μL)	819	839	1178	429	1829	552
CD4-t	345–2350 (/μL)	2313	1819	3789	2132	2162	1325
NK	210–1514 (/μL)	14.98	611	216	217	310	299
CD19-	240–1317 (/μL)	622	323	586	512	472	876
CD4/CD8	0.96–2.05 (/μL)	2.82	2.17	3.22	4.97	2.72	2.40
Beta-ᴅ-glucan (BDG)	<100, negative;	221	238	293	296	276	316
	100–200, suspected;						
	>200, positive						
GM test	<0.5	Negative	2.7	3.2	1.6	Negative	Negative
Microbiological cultures	–	*Candida guilliermondii*	*Aspergillus flavus*	*Aspergillus flavus*	*Lichtheimia corymbifera & Enterovirus*	*Aspergillus flavus & Chlamydia pneumoniae*	*Candida tropicalis*
Others	–	Neonatal lupus syndrome	Methylmalonic acidemia	Chronic granulomatous disease	–	–	–

GLU = glucose, HCT = hematocrit, HGB = hemoglobin, Hs-CRP = hypersensitive C-reactive protein, M = monocyte, NK = natural killer cell, PLT = platelet, PRO = protein, RBC = red blood cell, WBC = white blood cell.

Upon admission, all neonates were initiated on empirical treatment with ceftazidime and cefuroxime as the initial antibiotic regimen. Once the microbial culture results became available, the treatment strategies were promptly adjusted accordingly. Each patient underwent antifungal therapy for 3 to 4 weeks, during which repeated cultures, biochemical tests, and radiographic evaluations were conducted. Discharge was determined based on clinical symptom resolution and supportive examination results. No recurrences were observed during the 3-month follow-up period. Detailed information is provided in Table 1.

## 5. Discussions

Our investigation of CA-IFIs in neonates reveals distinct characteristics compared with the predominantly hospital-acquired invasive fungal infections (HA-IFIs) reported in the existing literature. Both CA-IFIs and HA-IFIs necessitate prompt diagnosis and targeted therapy. Notably, CA-IFIs exhibit unique clinical manifestations, pathogen profiles, and underlying comorbidities, underscoring their distinct nature.

A nationwide survey in Japan reported that *Candida* species were the most common pathogens in neonatal IFIs, accounting for 21 of 23 cases, with the remaining 2 cases being mucormycosis and unidentified pathogens.^[7]^ However, this study focused on HA-IFIs. A similar retrospective study in China, involving 223 neonates with IFI from January 2009 to December 2022, found that most neonates were born prematurely (gestational age <28 weeks), and *Candida albicans* accounted for 41.3% of cases.^[8]^ Another study also confirmed that invasive candidiasis is a significant cause of sepsis in neonatal intensive care units, particularly among preterm infants.^[9]^ In contrast, our study on CA-IFIs revealed no clustering of pathogens, suggesting broader environmental exposure or host-specific susceptibility. This contrasts with the homogeneous pathogen profiles typically seen in nosocomial outbreaks and highlights the need for expanded pathogen screening in community settings.

Unlike HA-IFIs, which often manifests with hypothermia, feeding intolerance, and hypotension,^[10]^ our cohort exhibited fever, cough, and respiratory distress without classic septic toxicity. The prolonged, irregular fever patterns (e.g., case 3), obvious respiratory symptoms, and absence of prematurity seems the dominant risk factors further distinguish CA-IFIs from HA-IFIs, where extreme prematurity and prolonged NICU stays are key drivers.^[11,12]^ Our patients did not display the classic signs of severe septic toxicity. Their fever lacked a clear temporal pattern and tended to persist for extended periods (e.g., case 3 experienced intermittent fever for a week after initiating antifungal therapy). Notably, all patients in our study had coughing of varying severity, which was a consistent feature throughout their illness.

Nonspecific laboratory markers, including elevated Hs-CRP, IgM, and CD4/CD8 ratio, were suggestive of nonspecific infections. Additionally, multiple nodular opacities on lung imaging studies are often mistakenly attributed to Staphylococcus aureus lung abscesses. In light of the initial findings, empirical antibiotic therapy was promptly initiated. Subsequently, positive results from the 1,3-β-ᴅ-glucan and GM tests prompted further pathogen identification. Metagenomic next-generation sequencing (mNGS) was performed and yielded definitive results within 2 days, while traditional fungal culture was conducted in parallel and provided confirmatory evidence within 3 to 7 days. Therapeutic strategies were refined promptly. Encouragingly, all patients in our cohort achieved complete recovery without any observed complications or disease recurrence. It is important to note that, given the limitations of fungal culture,^[13]^ all neonates underwent mNGS at the same time, and the results were consistent between both assays. This highlights the utility of 1,3-β-ᴅ-glucan and GM assays for early suspicion of fungal infection, followed by confirmation via mNGS, aligns with emerging diagnostic paradigms emphasizing multimodal approaches.^[14]^

A notable feature of our study was the consistent presence of co-occurring conditions among all pediatric subjects. Specifically, cases 1, 2, and 3 exhibited immune and metabolic dysfunctions, while Cases 4 and 5 were complicated by Enterovirus and *C pneumoniae* infections, respectively. This highlights that CA-IFIs are more closely associated with immune, metabolic, and genetic disorders (e.g., neonatal lupus, methylmalonic acidemia, *CYBB* mutations) rather than typical HA-IFIs risk factors such as central catheters or parenteral nutrition.^[15]^ Our findings are consistent with a large Chinese multicenter study that linked IFIs to congenital immunodeficiencies (e.g., *CYBB, CD40LG* mutations).^[16]^ The presence of CA-IFIs may indicate underlying undiagnosed genetic or immune defects. The concurrent detection of viral (Enterovirus) and atypical bacterial (*C pneumoniae*) pathogens in our cohort is uncommon in HA-IFIs. This co-occurrence raises questions regarding potential synergistic immunosuppression or unique shared environmental exposures in community settings.

Neonatal fungal encephalitis is rarely reported in the literature. However, a study investigating the correlation between neonatal IFIs and cranial MRI revealed that among 61 neonates with IFIs, abnormal signals were detected on T2-weighted (T2W1) and T1-weighted (T1W1) MRI images, with some patients also exhibiting meningeal hyperplasia.^[17]^ This suggests that a certain proportion of neonates with IFIs may have concomitant fungal encephalitis. Another study on neonatal invasive *Candida* infection reported that due to the insensitivity of cerebrospinal fluid tests and brain function imaging, all infected infants should be considered to have central nervous system (CNS) involvement.^[18]^ In our study, case 4 presented with dual fungal and viral CNS infection, highlights an underrecognized aspect of neonatal IFIs. Given that HA-IFIs studies often neglect CNS evaluation due to diagnostic limitations, our findings support the routine use of cerebrospinal fluid analysis and neuroimaging in suspected IFIs cases, in line with recent calls for comprehensive CNS assessment.^[19]^

Our study underscores CA-IFIs as a clinically and etiologically distinct entity from HA-IFIs, necessitating heightened suspicion in neonates with prolonged fever, respiratory symptoms, and comorbid immune-metabolic conditions. The strong link to genetic defects (e.g., *CYBB, RAG1* mutations) echoes findings in congenital immunodeficiency-associated IFI,^[16]^ suggesting that CA-IFI may warrant genetic testing even in the absence of classic immunodeficiency phenotypes.

The small sample size precludes definitive conclusions about pathogen prevalence or risk stratification. However, the concordance of mNGS and culture results supports the reliability of advanced diagnostics in resource-equipped settings. Larger multicenter studies are needed to validate our observations and explore the interplay between genetic susceptibility, environmental fungi, and co-infections in CA-IFIs.

## 6. Conclusion

This study illuminates the distinct clinical and etiological profiles of CA-IFIs in neonates, thereby challenging the traditional hospital-centric approach to neonatal IFIs research. Our findings underscore the need to focus on early genetic screening and immunological profiling in neonates with unexplained fungal infections, especially in resource-limited settings where diagnostic delays can exacerbate outcomes. Future research should prioritize elucidating environmental fungal exposures and host–pathogen interactions in community settings. By addressing these gaps, our work lays the foundation for personalized, preemptive management strategies for neonatal IFIs, with the ultimate goal of reducing morbidity and mortality in this vulnerable population.

## Author contributions

**Formal analysis:** Chunfang Gao.

**Funding acquisition:** Chunfang Gao.

**Investigation:** Xuwei Tao.

**Project administration:** Lingkong Zeng.

**Supervision:** Lingkong Zeng, Xuwei Tao.

**Writing – original draft:** Chunfang Gao, Li Wang.

**Writing – review & editing:** Xuwei Tao.
